# Optimization of CO_2_ Supply for the Intensive Cultivation of *Chlorella sorokiniana* IPPAS C-1 in the Laboratory and Pilot-Scale Flat-Panel Photobioreactors

**DOI:** 10.3390/life12101469

**Published:** 2022-09-21

**Authors:** David A. Gabrielyan, Boris V. Gabel, Maria A. Sinetova, Alexander K. Gabrielian, Alexandra G. Markelova, Natalia V. Shcherbakova, Dmitry A. Los

**Affiliations:** K.A. Timiryazev Institute of Plant Physiology, Russian Academy of Sciences, Botanicheskaya Street 35, 127276 Moscow, Russia

**Keywords:** *Chlorella*, biomass cultivation, carbon dioxide supply, carbon fixation rate, flat-panel photobioreactor, microalgae, scaling-up

## Abstract

Microalgae are increasingly being used for capturing carbon dioxide and converting it into valuable metabolites and biologically active compounds on an industrial scale. The efficient production of microalgae biomass requires the optimization of resources, including CO_2_. Here, we estimated the productivity of *Chlorella sorokiniana* IPPAS C-1 depending on CO_2_ concentrations and the ventilation coefficient of the gas-air mixture (GAM) in flat-panel photobioreactors (FP-PBRs) at laboratory (5 L) and pilot (18 L) scales. For the laboratory scale, the PBRs operated at 900 µmol quanta m^−2^ s^−1^ and 35.5 ± 0.5 °C; the optimal CO_2_ flow rate was estimated at 3 mL CO_2_ per 1 L of suspension per minute, which corresponds to 1.5% CO_2_ in the GAM and an aeration rate of 0.2 vvm. These parameters, being scaled up within the pilot PBRs, resulted in a high specific growth rate (µ ≈ 0.1 h^−1^) and high specific productivity (P_sp_ ≈ 1 g dw L^−1^ d^−1^). The principles of increasing the efficiency of the intensive cultivation of *C. sorokiniana* IPPAS C-1 are discussed. These principles are relevant for the development of technological regimes for the industrial production of *Chlorella* in flat-panel PBRs of various sizes.

## 1. Introduction

Intensive cultivation of microalgae is a promising method to capture and utilize the excess carbon dioxide [1,2]. As a result, the task of carbon footprint reduction with the simultaneous production of high-value products, theoretically, has been solved [3,4,5] and reflected in a solid number of publications [6,7,8,9,10,11,12,13,14,15,16,17,18,19,20,21,22,23]. It was demonstrated that, depending on a variety of parameters and cultivation conditions, a high carbon fixation rate (CFR) can be achieved [6,7]. The highest CFR value of 2.632 g L^−1^ d^−1^ was demonstrated for *Chlorella sp.* KR-1 [8]. However, this value was obtained in a small volume of suspension (50 mL) and at a high volumetric flow rate of CO_2_ (R_CO2_) of 0.01 vvm (volume of supplied gas per unit volume of growth medium per minute). Obviously, it is relatively easy to reach a high CO_2_ fixation rate in small-scale cultivation experiments. However, in this case, the biomass yield would be proportionally small, which makes no practical sense.

The CFR value is not much more informative or useful because it is calculated based on the ratio of 1.88 g CO_2_ per 1 g of dry weight of microalgal biomass [6] (pp. 124–125) and it does not consider the entire amount of CO_2_ supplied to a system (M_CO2_). A comparison of fixed amount of CO_2_ biomass and M_CO2_ allows for the estimatation of the efficiency of the gas-air mixture (GAM) supply and CO_2_ loss. An increase in the cultivation volume to obtain a higher biomass yield leads to an increase in the CFR value, while the efficiency of CO_2_ utilization decreases due to a number of challenges associated with scaling-up, ranging from energy consumption to the design of photobioreactors (PBRs) [7,10,11].

High productivity for most algal species is observed over a wide range of CO_2_ concentrations, from 0.038 to 10% (*v*/*v*), and the recommended values for the aeration rate (R_GAM_) are in the range of 0.1–1.0 vvm for closed PBRs [6,7]. The optimal values of both parameters vary depending on the algal strain and configuration of the PBRs. The important question is, what should be the combination of CO_2_ concentration in a GAM and the R_GAM_ in order to effectively convert CO_2_ into a significant amount of biomass during a relatively short period of cultivation?

An R_GAM_ of 0.025–1.0 vvm, at concentrations of 5% and 10% CO_2,_ is recommended as it is economically justified for intensive cultivation. Specifically, for flat-panel photobioreactors (FP-PBR) of an industrial scale, a value of 0.05 vvm was reported [12]. However, those conclusions were based solely on technical parameters and theoretical calculations for a 250 L PBR without any experimental data. Another report [14] demonstrated that an R_GAM_ below 0.1 vvm with 5% CO_2_ leads to a significant decrease in mass transfer in a FP-PBR, leading to the slow growth of the algae. It was concluded that the search for optimal conditions for the GAM supply must be carried out based on two parameters: the CO_2_ utilization efficiency (CUE) and specific productivity of the culture (P_sp_). The conditions of GAM supply include parameters such as the concentration of CO_2_ in a GAM; R_GAM_; GAM supply method; the diameter and trajectory of the bubbles; the geometry of the gas sprayer and the number of holes in it; the presence of baffles in a PBR, etc. [9,10,11,12,13,14,15,16,17,18,19]. All these parameters are important for determining the characteristics of the PBRs of various designs. In addition, the optimization of gas supply remains an urgent task for each individual algal strain.

The optimization of the GAM supply to a suspension of *Chlorella* had a significant impact on its productivity [15,16,17]. A P_sp_ of 0.4 g L^−1^ d^−1^ and a CUE of 28% were obtained for *Chlorella vulgaris* in an airlift FP-PBR (50 L) [15]. A CUE of 63% was reported for *Chlorella* sp. NCTU-2 in a porous centric-tube PBR, with a maximum P_sp_ of 0.6 g L^−1^ d^−1^ [16]. Moreover, a CUE of 81% was reached for *Chlorella sorokiniana* TH01 while a P_sp_ of 0.42 g L^−1^ d^−1^ was used in an outdoor FP-PBR (100 L) [17]. In the latter report, *C. sorokiniana* TH01 was defined as a producer of lutein from CO_2_ (up to 10 mg g^−1^).

Our previous studies on the cultivation of various strains of microalgae and cyanobacteria in the laboratory system made it possible to determine their technological ranges, which are suitable for the intensive cultivation of a wide range of photosynthetic microorganisms [20]. Those results provided the initial experimental data for the design of scalable flat-panel vertical PBRs with a high biomass yield [24] and allowed for the refinement of the technologies for the cultivation of photosynthetic microorganisms. The design of such PBRs and the applied technical solutions ensure high efficiency when using the light energy from artificial sources and high efficiency of CO_2_ utilization, excluding the contamination of the suspension and providing the possibility of stabilizing the cultivation parameters over a wide range. [10,11,21,22].

Here we determined the influence of the GAM supply on the specific productivity of *C. sorokiniana* IPPAS C-1. The strains of *Chlorella* are characterized by a high growth rate. The strain *C*. *sorokiniana* IPPAS C-1 was previously used for investigations into the parameters of industrial photobioreactors [24,25] and algal physiology [26,27]. We also estimated the CUE in newly assembled FP-PBRs. To construct the PBRs presented here, we used materials and products available on the market, which significantly simplified the assembly of these devices. The GAM was supplied to the PBRs with CO_2_ concentrations ranging from 0.36 to 7.75% and a corresponding R_GAM_ of 0.1 to 0.8 vvm to maintain constant R_CO2_. The results of the cultivation of *C. sorokiniana* IPPAS C-1 in the laboratory (5 L) and pilot PBR (18 L) were compared using equalized specific characteristics. The results of the work are relevant to the development of technological regulations for the industrial production of *C. sorokiniana* IPPAS C-1 in FP-PBRs of various sizes and volumes.

## 2. Materials and Methods

### 2.1. Microalgal Strain and Maintenance Conditions

The axenic strain of *Chlorella sorokiniana* IPPAS C-1 was obtained from the collection of microalgae and cyanobacteria IPPAS (K.A. Timiryazev Institute of Plant Physiology, RAS, Moscow, Russia). The axenic culture was maintained on slants of Tamiya agarized medium [23,24] in glass tubes at 22 °C under continuous illumination with cool white luminescent lamps of 30 μmol photons m^−2^ s^−1^. For the experiments, cells of *C. sorokiniana* were grown for 10–14 days in 300 mL Erlenmeyer flasks with 100 mL of ½ Tamiya modified medium [24] on an orbital shaker at room temperature under average illumination of 50 μmol photons m^−2^ s^−1^ from warm white light emitting diodes (LEDs). ½ Tamiya modified medium composition, g L^−1^: NaNO_3_—2.1; MgSO_4_·7H_2_O—1.25; KH_2_PO_4_—0.625; FeSO_4_·7H_2_O—0.009; EDTA—0.037; trace element solution (TES) 1 mL L^−1^; TES composition, g L^−1^: H_3_BO_3_—2.86; MnCl_2_·4H_2_O—1.81; ZnSO_4_·7H_2_O—0.222, MoO_3_·2H_2_O—0.018, NH_4_VO_3_—0.023.

All measurements of the irradiation level in the working volume of the PBRs and on the surface of the LED modules were recorded with a quantum meter LI-189 equipped with LI-190SA quantum sensor (LI-COR, Lincoln, NE, USA) in μmol photons m^−2^ s^−1^. Spectral composition of the light from LED was measured by LI-180 Spectrometer (LI-COR, Lincoln, NE, USA). Results of measurements were used to calculate the average irradiance on surfaces of PBRs.

### 2.2. Algal Pre-Culture for PBR Inoculation

The initial culture of *C. sorokiniana* IPPAS C-1 was grown aseptically in the laboratory system for intensive cultivation as described in [24]: in ½ Tamiya medium at 32 ± 0.6 °C under continuous illumination of 500 μmol photons m^−2^ s^−1^ provided by warm white, blue, and red LEDs (description of LED module and spectral composition of the light are presented in the supplementary material in Figure S1). Culture mixing and aeration were achieved by bubbling with sterile GAM that contained 1.5–2% CO_2_. The resulting culture with a dry biomass concentration (ρ) of 2–5 g dw L^−1^ was used as inoculum for the next stage.

### 2.3. Flat-Panel PBRs

#### 2.3.1. Principal Scheme of Experiments

**Figure 1:**
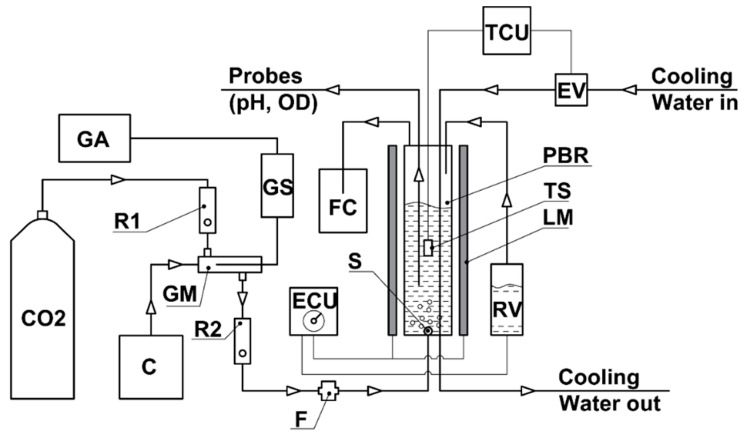
Principal working scheme of the experimental unit. CO_2_—carbon dioxide cylinder; GA—gas analyzer; C—air compressor; R1—CO_2_ rotameter; R2—gas-air mixture rotametr; F—filter; GS—gas analyzer sensor; FC—foam container; GM—gas-air mixer; ECU—electric control unit; TCU—temperature control unit; EV—electromagnetic valve; PBR—photobioreactor; S—gas-air mixture sprayer; RV—reserve water (medium) volume; TS—temperature sensor; LM—LED modules; OD—optical density; pH—hydrogen ion exponent.

Both FP-PBRs (FP-5 and FP-18) employed similar principal working schemes (Figure 1).

#### 2.3.2. Design of FP-PBR with a Working Volume of 5 L (FP-5)

**Figure 2:**
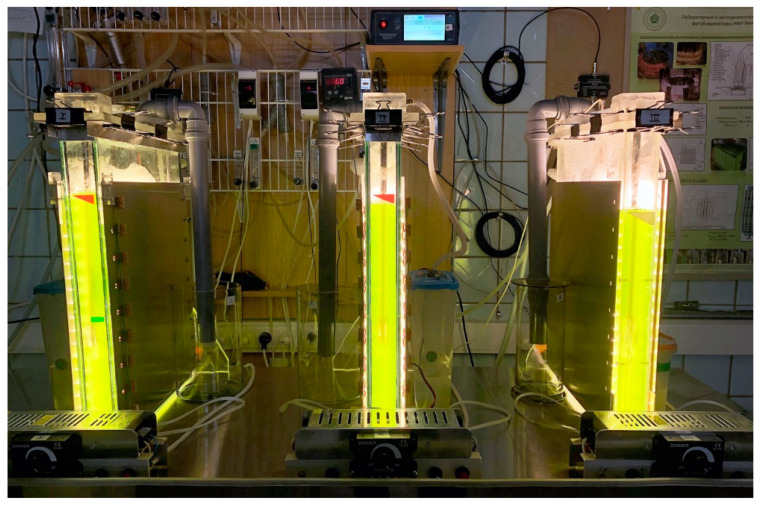
Photograph of the three flat-panel laboratory photobioreactors FP-5 filled with a suspension of *Chlorella sorokiniana* IPPAS C-1.

The PBR, with a working volume of 5 L, consists of a glass aquarium with an internal volume of 361 mm × 40 mm × 460 mm, submersible module with cooling and hot water supply systems, and two LED modules, consisting of 18 rows of LED strips (Figure S1). The average irradiance on the light-receiving surface of the PBR was 900 µmol m^−2^ s^−1^. Modules were installed close to the sides of the reactor. Three PBRs (of this construction type) have been used in the current work. Figure 2 presents a photo of the reactors. The operation of PBR FP-5 is demonstrated in Video S1.

#### 2.3.3. Design of FP-PBR with Working Volume of 18 L (FP-18)

**Figure 3:**
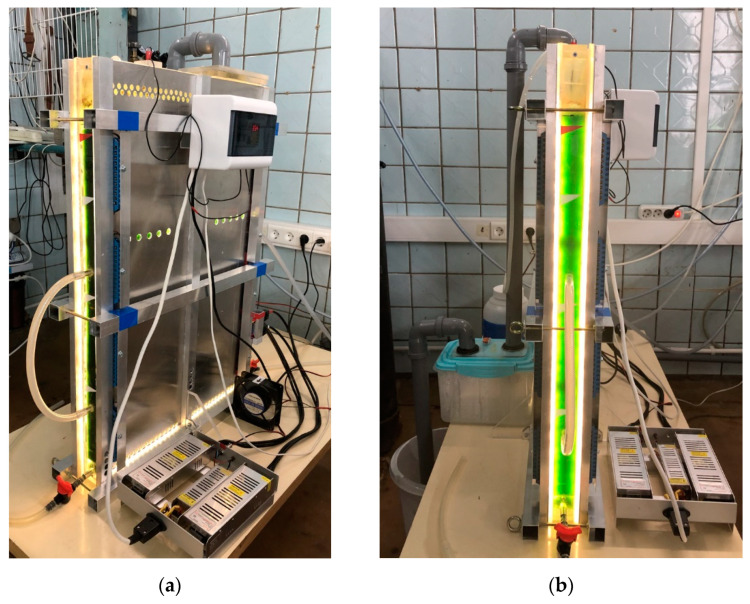
Photograph of the flat-panel pilot photobioreactor FP-18 filled with a suspension of *C. sorokiniana* IPPAS C-1: (**a**) general view; (**b**) reactor’s end face.

The PBR with a working volume of 18 L consists of a demountable container formed by plane-parallel translucent walls and two LED modules (Figure 3). PBR working volume dimensions: 600 mm × 40 mm × 800 mm. The LED modules are adjacent on both sides of the container, directly to the translucent walls, and are equipped with radiators to cool the LEDs. The design of the modules allows to discretely change the number of working LEDs and their distribution over the irradiated area. The operation of PBR FP-18 is demonstrated in Video S2.

#### 2.3.4. Main Parameters of the PBRs

The geometrical and light characteristics of the PBRs used in this study are presented in Table 1. The main difference between FP-5 and FP-18 is their size. FP-18 is 1.74× higher and 1.67× longer than FP-5, but they have an identical light path (40 mm), identical irradiation level on the light receiving surface, and identical gas sprayers at the bottom of the reservoirs.

Starting biomass concentrations in both PBRs were also similar (ρ_0_ = 0.14 ± 0.04 g dw L^−1^), with an average pH level of 6.0 ± 0.15. Thereby, the comparative characterization of these PBRs could be accepted as appropriate. Scaling-up the cultivation was performed using combinations of 1.5% or 2% CO_2_ in a GAM with an of R_GAM_ 0.2–0.23 vvm.

Water for filling the PBRs was purified by six-stage reverse osmosis system AP-800DIR-400 (AquaPro Industrial Co., Ltd., Nanking E. Rd., Taipei, Taiwan). All containers, hoses, filters, and liquids were either sterilized by autoclaving or treated with hot steam and 70% ethanol.

#### 2.3.5. Gas-Air Mixture Supply

The GAM was supplied to the suspension through an aquarium sprayer with 8 holes with a step of 10 mm (∅ 1 mm; Hailea HL-AC04), placed at the bottom of each PBR. In the FP-5, the sprayer length was 300 mm, while for FP-18, it was 500 mm. Pure CO_2_ from a cylinder was mixed with air by a compressor in a mixing unit. Then, passing through rotameters, CO_2_ concentration sensors and filters (Millex-FH Filer Syringe Filter Unit, 0.45 μm or 0.2 μm), the mixture eventually passed into the suspension through the sprayers. In PBR FP-5, the volumetric flow rate of the supplied mixture was 0.5, 1, 2, or 4 L min^−1^; and in PBR FP-18, it was 3.7 or 8.3 L min^−1^. Concentration of CO_2_ and the R_GAM_ were controlled by rotameters R1 and R2 (Figure 1). Different aeration ratios define bubbles distribution in the PBRs (Video S3).

The pipes installed on the upper faces of the PBR are designed to remove foam and release the waste GAM, including the oxygen generated during photosynthesis. The design of the GAM release system eliminates the contamination of the suspension by possible contaminating microorganisms.

#### 2.3.6. Temperature Control System

Thin-walled stainless-tube heat exchangers were installed perpendicular to the suspension flow in the PBR. The FP-5 has one tube, while FP-18 has two tubes. Temperature control of the suspension is carried out due to the periodic flow of the coolant through the heat exchanger tube. The automatic regulation of the cultivation temperature is controlled by a signal from a temperature sensor located in the internal volume of each PBR.

The temperature conditions (35.5 ± 0.5 °C) in all experiments were identical. The choice of this temperature is due to the maximum growth rates of *C. sorokiniana* IPPAS C-1 during the first three days (Table S1) and savings in energy consumption for the cooling.

### 2.4. Growth Characteristics

Growth characteristics were calculated as described before [24]. The pH level of each sample taken for measurement of optical density of the suspension was estimated with a Mettler Toledo SevenEasy pH meter equipped with an Inlab 413 electrode.

The carbon dioxide utilization efficiency (CUE) was calculated as (%):

(1) $$CUE=1.88\left(M_{PBR}-M_{0}\right)/M_{CO2}$$

where 1.88 is a coefficient to recalculate an amount of fixed CO_2_ in a microalgae cell based on the typical microalgal molecular formula, CO_0.48_H_1.83_N_0.11_P_0.01_ [6]; (*M_PBR_* − *M*_0_)—accumulated weight of dry biomass in the working volume of the PBR, and M_CO2_ is the mass of CO_2_ which was supplied through the PBR. *M_CO_*_2_ was calculated as total CO_2_ volume multiplicated by ρ_CO2_ = 1.82 g L^−1^ (20 °C, 10^5^ Pa). CUE is the ratio of the total supplied CO_2_ conversion into biomass that depends not only on the capability of an algal strain, but also on the design parameters of a PBR.

### 2.5. Biochemical Composition

Samples of 7–15 mg (of the lyophilized biomass from the final drain) were used for dry mass estimation, protein, and starch content measurements. The samples for dry weight estimation were incubated overnight at 80 °C in pre-weighted plastic microtubes and then weighed. The detailed procedures of protein and starch extraction and determination of their concentration are described in [20]. Briefly, the total protein concentration was estimated with BCA assay, and the starch concentration was measured using the phenol-sulphuric method. All measurements were carried out in triplicate.

### 2.6. Statistics

The graphs of the growth curves represent the mean values for two biological replicates and their mean deviations. The curves without bars represent mean values for technical replicates made in the individual experiments.

## 3. Results

### 3.1. Selection of CO_2_ Concentration in a GAM

The first step of this work was to determine the optimal level of R_CO2_ at a fixed level of R_GAM_ in the FP-5 photobioreactors. The initial level of GAM flow was 1 L min^−1^, which corresponds to an R_GAM_ of 0.2 vvm and falls into the range of the optimal values determined for other FP-PBRs [7,12,14]. The experiments were carried out in parallel in three individual PBRs. To study the effect of CO_2_ supply, we used only the actively growing cultures which were not limited by other factors, e.g., macronutrients. A significant decline in specific growth rate was observed on the 4th day of cultivation, meaning that the experiment duration was 3 days.

Volumetric GAM CO_2_ concentrations of 1%, 1.5%, 2%, and 4% were tested. Experiments with concentrations of 1%, 1.5%, and 2% were carried out in two biological replications. At the end of the cultivation, the pH of the culture medium was nearly identical (pH = 8.1 ± 0.2) in all variants examined.

**Figure 4:**
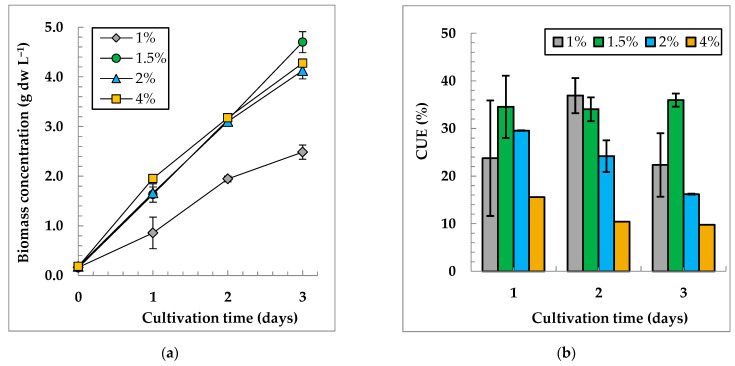
Biomass concentration dynamics (**a**) and CO_2_ utilization efficiencies (**b**) of *C. sorokiniana* IPPAS C-1 in FP-5 under different GAM CO_2_ concentrations and a constant R_GAM_ = 0.2 vvm. I_ave_ = 900 µmol m^−2^ s^−1^; T= 35.5 ± 0.5 °C.

In our experimental conditions, the following dependences of the dry biomass concentration of *C. sorokiniana* IPPAS C-1 on CO_2_ concentrations in a GAM, were revealed (Figure 4).

Figure 4a shows that the variants with 1.5%, 2%, and 4% CO_2_ turned out to be almost identical in terms of biomass accumulation, while the variant with 1% CO_2_ had the worst growth. On the third day of cultivation, the reliable differences in the biomass concentration became visible in the variant with 1.5% CO_2_. In the culture grown at 1.5% CO_2_, the CUE (35 ± 2% and P_sp_ of 1.51 ± 0.07 g dw L^−1^ d^−1^) was higher than that for other variants (Figure 4b, Table S2). Therefore, for further investigation, we chose the value of R_CO2_ = 0.003 vvm, which corresponds to 1.5% CO_2_ at 0.2 vvm.

### 3.2. Optimization of R_GAM_ and Concentartion of CO_2_

By fixing the R_CO2_ in the vicinity of 0.003 vvm, we varied the combinations of the R_GAM_ (vvm) and the GAM CO_2_ concentration (%): 0.77 vvm and 0.36%; 0.4 vvm and 0.75%; and 0.1 vvm and 3%. The experiments with the first two combinations were carried out for two biological replications. At the end of the cultivation, the pH level was lowest in the variant with 7.9% CO_2_ (pH = 7.7 ± 0.13) and highest in the variant with 0.36% CO_2_ (pH = 8.7). In all other variants, the average pH values were in the range of 7.9–8.4.

**Figure 5:**
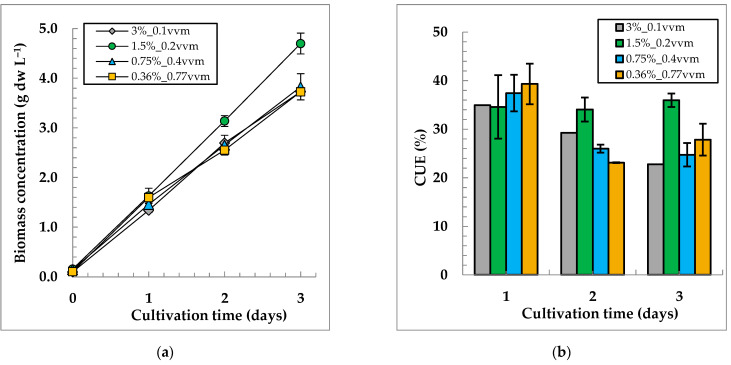
Biomass concentration dynamics (**a**) and CO_2_ utilization efficiencies (**b**) of *Chlorella sorokiniana* IPPAS C-1 grown in the FP-5 under different GAM supply conditions and a constant R_CO2_ = 0.003 vvm. I_ave_ = 900 µmol m^−2^ s^−1^; T = 35.5 ± 0.5 °C.

The obtained dependences for the dry biomass concentration of the GAM supply conditions and CUE values at a fixed R_CO2_ are presented in Figure 5.

Additionally, the experiments with a higher R_CO2_ = 0.008 vvm were carried out for one biological replicate (Figure S3). The following combinations of R_GAM_ and GAM CO_2_ concentration were used: 0.8 vvm and 0.94%; 0.4 vvm and 2%; 0.2 vvm and 4%; and 0.1 vvm and 7.73%. All results are summarized in as comparisons in Table S2.

The results show that, at a fixed R_CO2_ = 0.003 vvm, the variant with 1.5% CO_2_ and an R_GAM_ = 0.2 vvm remains preferable compared to the other combinations. A comparison of this variant to the group of experiments performed at N R_CO2_ = 0.008 vvm shows that the increase in R_CO2_ does not improve the final biomass concentration and results in 3 times lower values for CUE.

The experiments with an increased R_GAM_ = 0.45 vvm and the same CO_2_ concentration (of 1.5% (R_CO2_ = 0.007 vvm)) were carried out for two biological replicates to verify the chosen combination. Table S2 demonstrates that the doubling of R_CO2_ does not give a noticeable increase in biomass yield, which increased by only 8.6%, while the CUE value decreased two-fold.

### 3.3. Scaling-Up the Optimal Conditions

**Figure 6:**
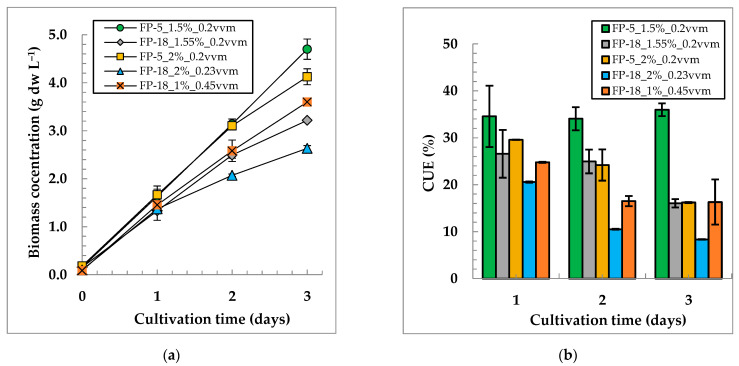
Biomass concentration dynamics (**a**) and CO_2_ utilization efficiencies (**b**) of *C. sorokiniana* IPPAS C-1 in FP-5 and FP-18 under identical GAM supply conditions. I_ave_ = 900 µmol m^−2^ s^−1^; and T = 35.5 ± 0.5 °C.

The scaling-up of the cultivation was performed using the chosen optimal combination of 1.5% and an R_GAM_ = 0.2 vvm and the combination of 2% CO_2_ with an R_GAM_ of 0.2 vvm. Figure 6 shows a comparison of the growth characteristics for *C. sorokiniana* IPPAS C-1 in FP-5 and FP-18. The obtained results confirm a preferable expenditure of 1.5% instead of 2% CO_2_ in FP-18. In these experiments, the pH level varied in the range of 7.9 to 8.3 units.

Notably, the direct transfer of specific cultivation parameters from a small to a large scale does not ensure a proportional increase in biomass yield. After 3 days of cultivation, the biomass yield for FP-18 was 57 ± 0.4 g dw, while for FP-5, it was 22.6 ± 1 g dw. Thus, a 3.6-fold increase in PBR volume gives only a 2.5-fold increase in biomass yield. The specific productivity of FP-18 turned out to be lower than that of FP-5 (1.1 ± 0.01 *vice* 1.5 ± 0.07 g dw L^−1^ d^−1^).

We tried to proportionally increase the biomass yield and CUE in FP-18, with an increase in R_GAM_ (0.45 vvm) and an R_CO2_ = 0.0045 vvm and 1% CO_2,_ tested. The results revealed that, on the third day of cultivation, the increase in the R_GAM_ enhanced the biomass yield and CUE (Figure 6, Table S2).

### 3.4. Effect of CO_2_ Concentartion and Aeration Rate on Biomass Composition

*C. sorokiniana* IPPAS C-1 is mainly a source of protein and starch, so the content of these compounds was estimated in the cells grown under different regimes of GAM supply (Table 2). The protein content was in the range of 19–45% for d.w., while the starch content was in the range of 11–54%. The protein and starch contents were inversely related to each other. Direct dependence of biochemical composition on GAM supply conditions was not found. At the same time, the clear dependence of cell biochemistry on biomass concentration can be seen; in the cultures with a higher biomass concentration, the cells had more starch and less protein and vice versa.

## 4. Discussion

### 4.1. GAM Supply Conditions

The regime of GAM supply to a suspension of microalgae significantly affects growth and biomass accumulation. This becomes evident when the parameters, such as the concentration of CO_2_ in the GAM or aeration rate, are compared separately [8,9,12]. In these cases, the changes in the volume flow of the supplied CO_2_ may be critical for the growth of the culture. In combination with the appropriate irradiation, this parameter determines the maximum level of productivity [10,22]. Limiting the experiments to only one level of irradiation, we deliberately limited the growth potential of an algal culture. For the irradiance level of 900 µmol m^−2^ s^−1^ and aeration rate of 0.2 vvm, the saturation level of CO_2_ concentration for the growth of *C. sorokiniana* IPPAS C-1 was determined at 1.5%. Any increase in CO_2_ concentration in the GAM had no significant impact on the biomass yield. The optimal conditions of cultivation provided a reasonable yield of biomass (P_sp_ = 1.51 ± 0.07 g dw L^−1^ d^−1^, which is close to the maximum of 1.64 ± 0.02 g dw L^−1^ d^−1^) and a relatively high CUE of 35%. Higher CUE values can be reached by reducing the R_GAM_ and, accordingly, supplying smaller volumes of CO_2_. The latter, however, results in growth retardation and a lower biomass yield at the end of the cultivation cycle. In this way, CUE values of 81% have been achieved in *C. sorokiniana* TH01 with the highly reduced specific productivity of 0.42 g dw L^−1^ d^−1^ [17], which is three times less than those achieved in our work.

The insufficient supply of CO_2_ (e.g., ≤1% CO_2_) or absence of proper mixing in the suspension (e.g., ≤0.1 vvm R_GAM_) leads to foaming and to the formation of a noticeable sediment of algal cells at the bottom part of the PBRs (Figure S3). High values of R_GAM_ (0.4–0.8 vvm) caused the formation of a foam, while a decrease in R_GAM_ (0.1 vvm) caused the sedimentation of the cells. Both phenomena should be avoided during intensive biomass cultivation. The cell sedimentation problem can be solved by choosing the proper mixing conditions.

In the variants with 1.5–2% CO_2_ and an R_GAM_ in the vicinity of 0.2 vvm, we observed less cell sedimentation with low foaming intensity, when compared to the other variants. Figure S4 shows a photograph of the foam containers with the escaped cells, and the effect of aeration rate on the biomass lost volume in a FP-5. At an R_GAM_ of 0.1 vvm, less than 10 mL of lost biomass volume was accumulated in the foam container, while at an R_GAM_ of 0.8 vvm, 0.5 L (which is 10% of the FP-5 working volume) accumulated by the end of the cultivation cycle. In fact, the “escaped” or lost biomass may comprise a significant portion of the yield that depends on the intensity of the foaming process, which, in turn, may cause a noticeable decrease in the specific growth rate and productivity of an algal culture. The study of the parameters and conditions of active foaming can provide additional information on identifying the optimal conditions for algal cultivation. The foaming ability itself may be of interest to the food industry for the creation of new consumption products based on microalgae [28].

### 4.2. Enhancing Productivity and Carbon Dioxide Utilization

While maintaining the considered parameters for the GAM supply, it is possible to increase both CUE and productivity. This can be carried out by selecting the size and trajectory of the GAM bubbles, installing baffles or blades for culture mixing [9,19,29,30]. In this way, the yield of the *C. pyrenoidosa* was increased from 1.6 to 2.9 g dw L^−1^ [29]. The physics of the gas-liquid mass transfer processes require the use of a complex mathematical model in conjunction with carefully designed experiments [31,32,33]. The application of modern engineering calculation systems allows for the selection of the optimal sprayer shapes and promotes more detailed studies of the influence of the GAM supply on the growth characteristics of microalgae for each specific PBR and algal strain.

### 4.3. Scaling-Up the Optimal Conditions

The scaling-up of the cultivation parameters from 5 L to 18 L appeared relatively successful. However, in FP-18, the final biomass yield and CUE dropped on average by 30% and 35%, respectively, when compared to FP-5. Earlier comparisons of the performance of similar FP-PBRs, which differed in height or length, demonstrated that the mass transfer between the gaseous phase and the liquid algal suspension is directly proportional to the height and inversely proportional to the length of the PBR [14]. Thus, an increase in the longitudinal dimensions of the reactor may lead to a decrease in mass transfer and in the intensity of the mixing. This observation has been confirmed here, when a doubled R_GAM_ of 0.45 vvm with 1% CO_2_ was compared to 2% CO_2_ and an R_GAM_ of 0.23 vvm (Figure 6, FP-18_1%_0.45 vvm curve). The increase in the R_GAM_, keeping a constant R_CO2_ value, noticeably enhanced the growth and biomass yield. Finally, scaling up the cultivation parameters requires the application of a transfer coefficient as a certain multiplier.

### 4.4. The Effect of a GAM Supply on Protein and Carbohydrate Composition of the Biomass

The biomass obtained under various GAM supply regimes (except the last lane in Table 2) had a similar biochemical content. Therefore, we conclude that there was no direct effect of the studied GAM supply on the biochemical composition of the *C. sorokiniana* IPPAS C-1 cells. The apparent difference might be due to the enhanced aeration rate itself, or due to the influence of the GAM supply on the growth rate. On the third day of cultivation, when the samples were taken, the cultures had different biomass concentrations and were in different growth stages. The highest protein content is usually observed in exponentially growing cells [20], and exponential growth is possible when the cells are not limited in their light and nutrients [34]. Considering that all cultures were grown in the same medium and under the same average irradiance, the cultures with a lower biomass concentration were less limited with light and nutrients and, thus, were enriched in proteins at the expense of starch. The fast-growing culture in the last variant (R_GAM_ = 0.8 vvm; GAM CO_2_ concentration 0.98%) most probably reached the late linear phase of growth and was limited in its light and nitrogen supply, so the cells started to accumulate starch at the expense of protein.

For practical application, it is necessary to consider the growth phase of the culture, as protein-rich biomass can be used in food and feed production, while cells with a high starch content can be used for further fermentation and biofuel production. To keep a high protein content in fast-growing cultures, it is necessary to increase the amount of nitrogen in the medium. It is important to note that the scaling-up from 5 L to 18 L did not cause any significant effects on the biochemical composition of *Chlorella* biomass.

## 5. Conclusions

The regime of GAM supply to a suspension of microalgae significantly affects their growth and biomass accumulation. An insufficient CO_2_ supply, and too high of an R_GAM_, lead to foaming, while the absence of the proper mixing of the suspension caused the formation of noticeable sediments of algal cells in the bottom of the PBRs. It is important to avoid these side effects during intensive biomass cultivation. During the cultivation of *C. sorokiniana* IPPAS C-1 at 900 µmol m^−2^ s^−1^ and 35.5 ± 0.5 °C, the optimal GAM supply condition was determined to be 3 mL CO_2_ per 1 L of suspension per minute, which corresponds to an aeration rate of 0.2 vvm and GAM CO_2_ concentration of 1.5%. These optimal conditions for cultivation provided a reasonable yield of biomass (P_sp_ = 1.51 ± 0.07 g dw L^−1^ d^−1^) and a relatively high CUE of 35%. Any further increase in CO_2_ concentration in the GAM had no significant impact on the biomass yield.

The scaling-up of the cultivation parameters from 5 L to 18 L appeared to be relatively successful; even the final biomass yield and CUE decreased by ~30% and 35%, respectively, in FP-18. The increase in R_GAM_, keeping a constant R_CO2_, promoted growth and increased the biomass yield with rather similar biochemical characteristics.

The principles for increasing the efficiency of the intensive cultivation of *C. sorokiniana* IPPAS C-1 described above are relevant for the development of technological regimes for the industrial production of *Chlorella* in flat-panel PBRs of various sizes.

## Figures and Tables

**Table 1:** Main parameters of the FP-PBRs used in this study.

**PBR**	**Light Path, mm**	**Working Volume, L**	**Suspension Level, mm**
FP-5	40	5	370
FP-18	40	18	700
**PBR**	**Average Irradiation** **E_e_, μmol Photons m^−2^ s^−1^**	**The Ratio of the Illuminated Surface to the Volume SA/V, m^2^ m^−3^**	Specific Power Consumption * W_sp_, W L^−1^
FP-5	900	50.3	23
FP-18	900	46.7	22

*—electric power consumption of the PBRs used for the light system and ventilation of GAM.

**Table 2:** Starch and protein content in *C. sorokiniana* IPPAS C-1 cells grown for 3 days under different GAM supply conditions.

Variant	Starch, % of d.w. *	Protein, % of d.w. *	Final Biomass Concentraion, g dw L^−1^
FP-5 R_GAM_ = 0.1 vvm GAM CO_2_ concentration 7.9% R_CO2_ = 0.008 vvm	11 ± 2.7	41 ± 4.3%	2.7
FP-18 R_GAM_ = 0.43 vvm GAM CO_2_ concentration 1.02% R_CO2_ = 0.004 vvm	14 ± 1.9	45 ± 2.3	3.5
FP-5 R_GAM_ = 0.2 vvm GAM CO_2_ concentration 2.04% R_CO2_ = 0.004 vvm	20 ± 0.6	38 ± 8.6	4.0
FP-5 R_GAM_ = 0.2 vvm GAM CO_2_ concentration 4% R_CO2_ = 0.008 vvm	18 ± 1.7	38 ± 7.8	4.3
FP-5 R_GAM_ = 0.8 vvm GAM CO_2_ concentration 0.98% R_CO2_ = 0.008 vvm	54 ± 11.7	19 ± 2.9	4.9

* Mean values and their standard deviations are shown.

## Data Availability

Not applicable.

## Supplementary Materials

The following supporting information can be downloaded at: https://www.mdpi.com/article/10.3390/life12101469/s1, Figure S1: LED parameters and the spectral composition of the light; Figure S2: Biomass concentration (a) and CO_2_ utilization efficiency (b) of *C. sorokiniana* IPPAS C-1 in FP-5 under different GAM supply conditions and constant parameters: I_ave_—900 µmol m^−2^ s^−1^; T—35.5 ± 0.5 °C; R_CO2_—0.008 vvm; Figure S3: Photographs of the foam (a) and the sedimented culture (b) in FP-5 PBR; Figure S4: The effect on cultural volume lost by foaming process: (a)—foam containers with lost biomass under different R_GAM_ (0.1, 0.4, 0.8 vvm) and the same R_CO2_ of 0.003 vvm; (b)—dependence of lost volume on the aeration rate (R_GAM_); Table S1: Growth characteristics of *C. sorokiniana* IPPAS C-1 cultures grown for 3 days at different temperatures in laboratory system for intensive cultivation. R_GAM_ = 1 vvm, GAM CO_2_ concentration 1.2–1.8%, I_ave_ = 400 µmol m^−2^ s^−1^; Table S2: Comparative table of growth characteristics of *C. sorokiniana* IPPAS C-1 in FP-5 and FP-18 during 3 days of cultivation; Video S1: The operation of PBR FP-5; Video S2: The operation of PBR FP-18; Video S3: Video of bubble flows distributions in dynamic under different aeration rates in PBR FP-5.

## Abbreviations

The following abbreviations are used in this manuscript:

µ	specific growth rate
CFR	carbon dioxide fixation rate
CUE	cardon dioxide utilization efficiency
g dw	gram of dry weight
GAM	gas-air mixture
IPP RAS	Institute of Plants Physiology of Russian Academy of Sciences
LED	light emitting diode
ρ	dry biomass concentration
ρ_0_	starting dry biomass concentration
ρ_fin_	final dry biomass concentration
M_CO2_	mass of total supplied CO_2_
M_0_	starting weigh of dry biomass in PBR after inoculation
M_PBR_	weight of dry biomass in the working volume of PBR at the moment
PBR	photobioreactor
PBR FP	flat-panel photobioreactor
P_sp_	specific productivity
R_GAM_	GAM aeration rate
R_CO2_	CO_2_ aeration rate
T_dbl_	biomass doubling time
V_PBR_	working volume of PBR
vvm	volume of sparged gas per unit volume of growth medium per minute
