# Supplementary material for: Optimization of CO2 Supply for the Intensive Cultivation of Chlorella sorokiniana IPPAS C-1 in the Laboratory and Pilot-Scale Flat-Panel Photobioreactors
Source: Life (Basel). 2022 Sep 21;12(10):1469. doi: 10.3390/life12101469 (PMC9605657; doi:10.3390/life12101469)
Supplement: Supplementary file 1 [file life-12-01469-s001.zip › Supplementary Figures and Tables.pdf]

LED properties

**Type**	SMD2835
**Number (LED m^{-1})**	120
**Power (W m^{-1})**	9.6
**Color**	Warm white
**Color Temperature (K)**	3000
**Input Voltage (V)**	12
**CRI**	>80
**Ingress Protection Rating**	IP20

Spectral composition of the LED light

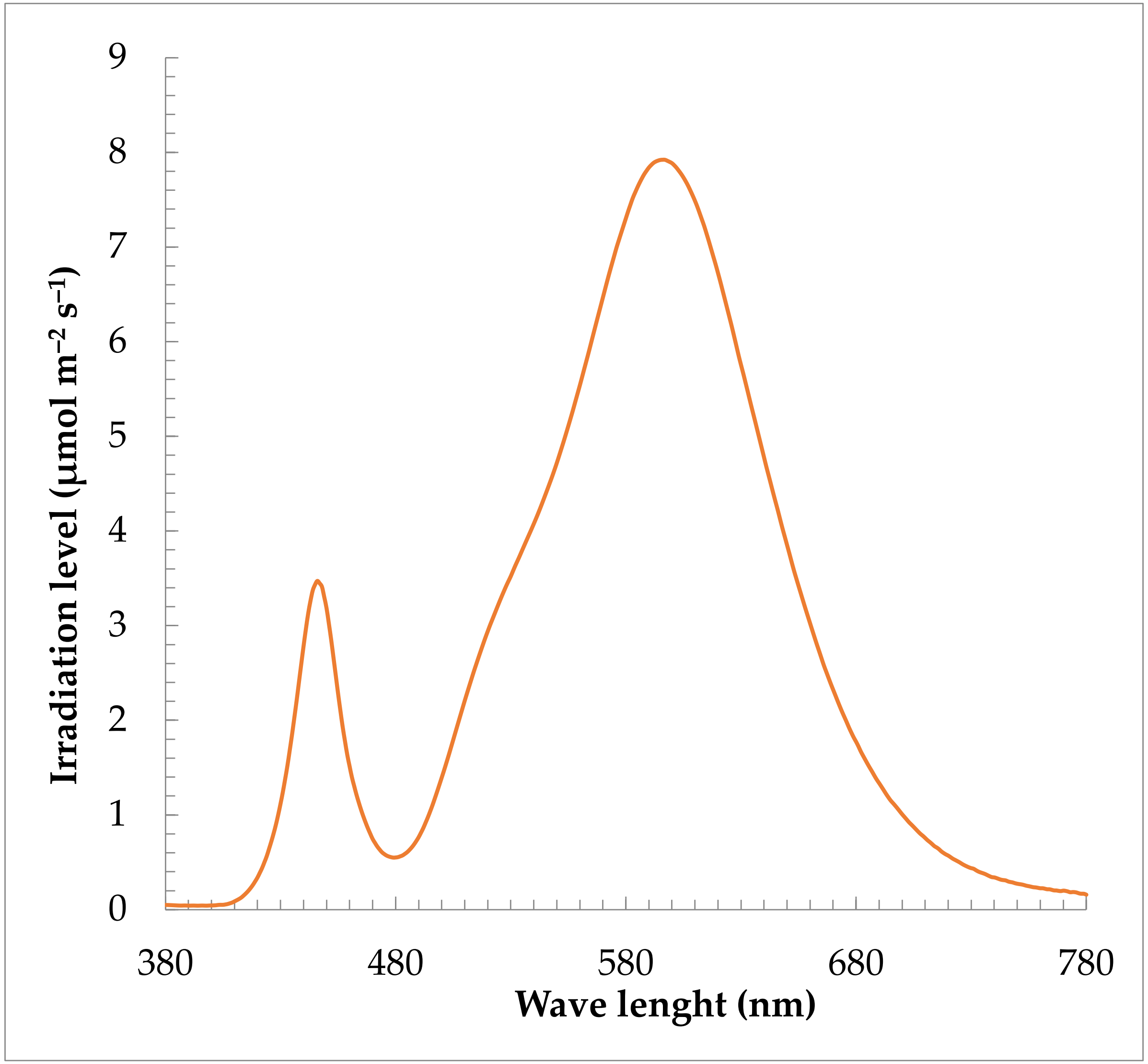

Figure S1. LED parameters and the spectral composition of the light.

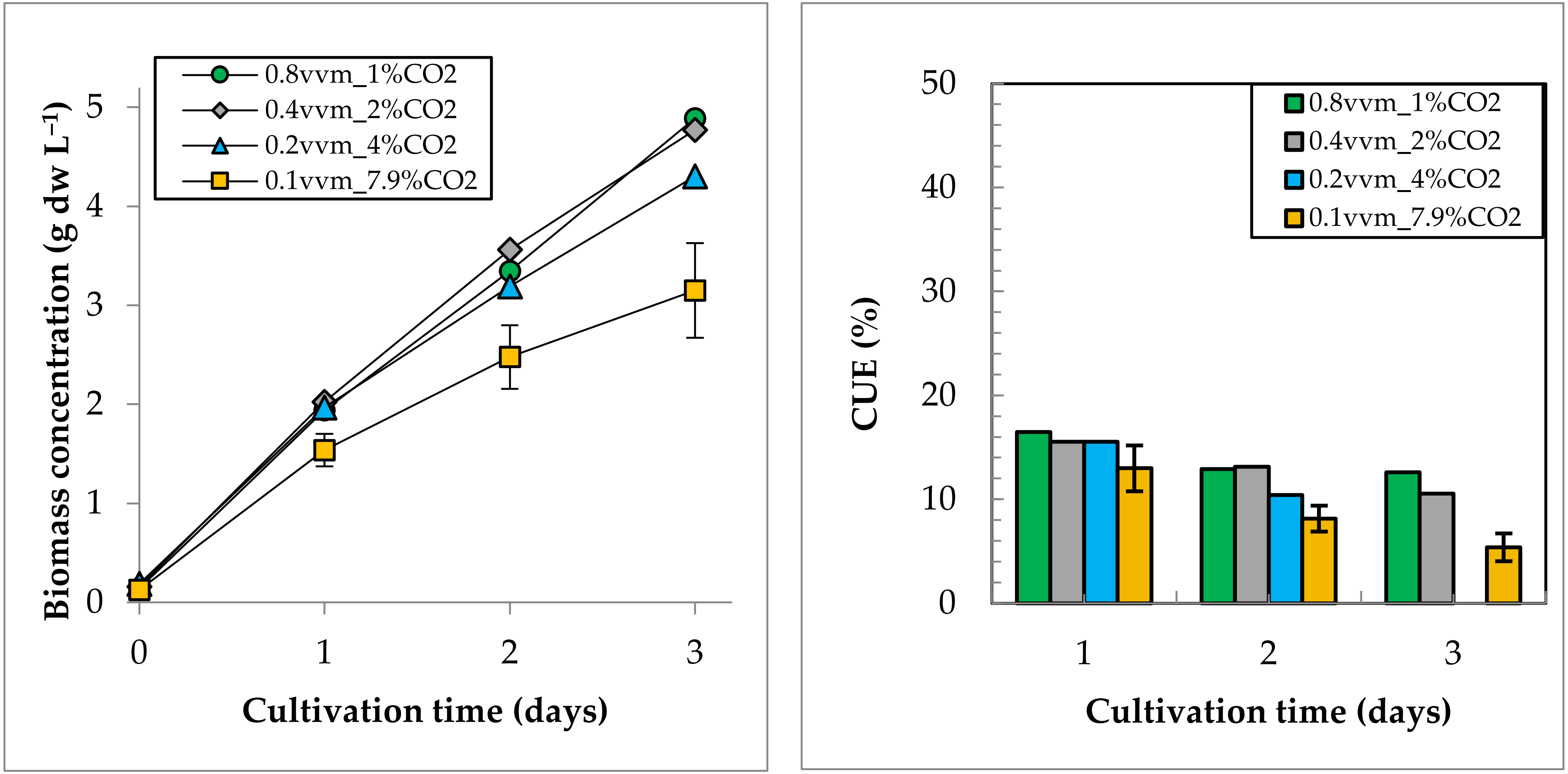

(a) **(b)**

Figure S2. Biomass concentration (a) and CO_2 utilization efficiency (b) of *C. sorokiniana* IPPAS C-1 in FP-5 under different GAM supply conditions and constant parameters: I_{ave} – 900 µmol m^{-2} s^{-1} ; T – 35.5±0.5 °C; R_{CO2} – 0.008 vvm.

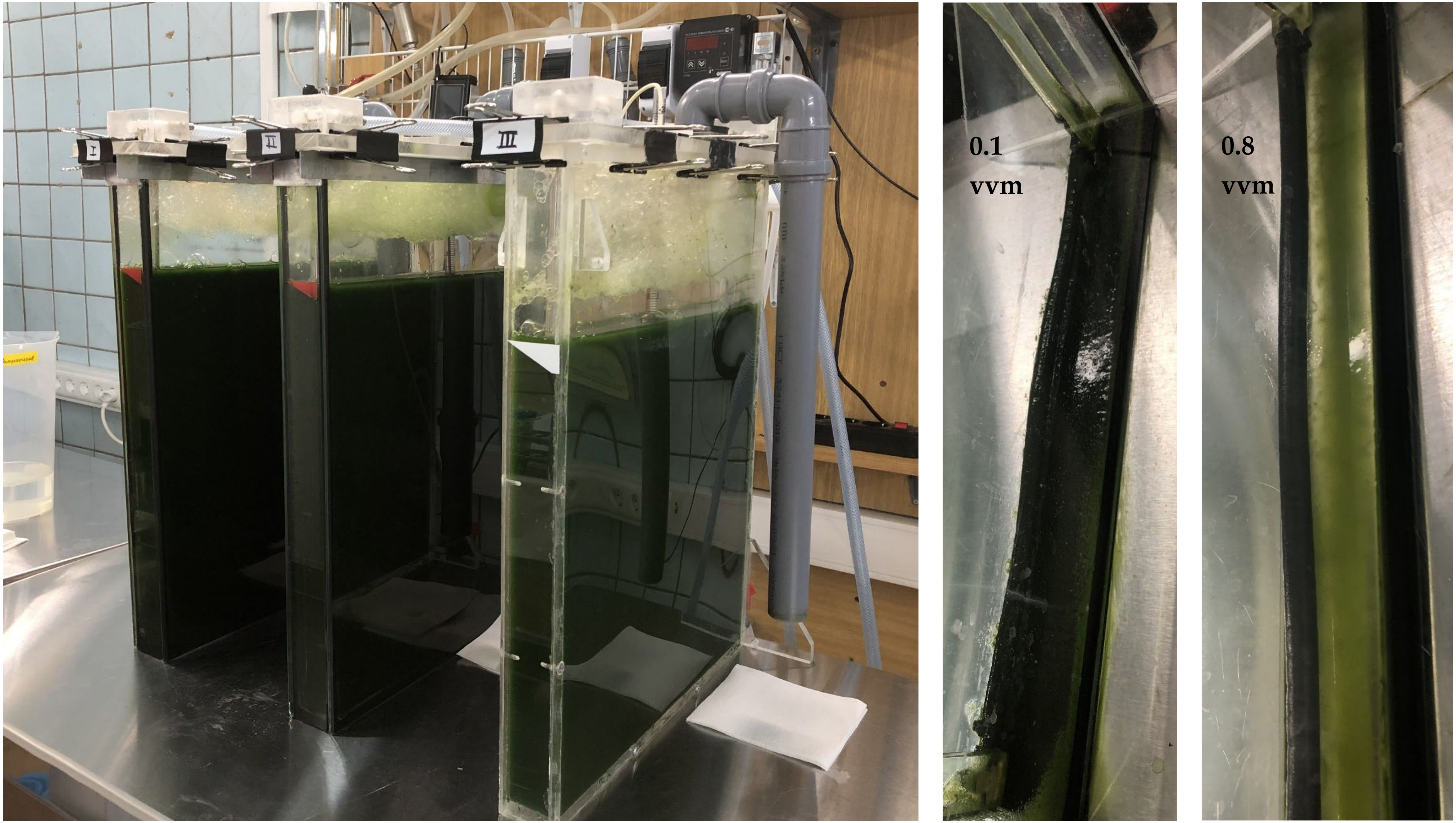

(a) (b)

Figure S3. Photograph of the foam (a) and the sedimented culture (b) in FP-5 PBR

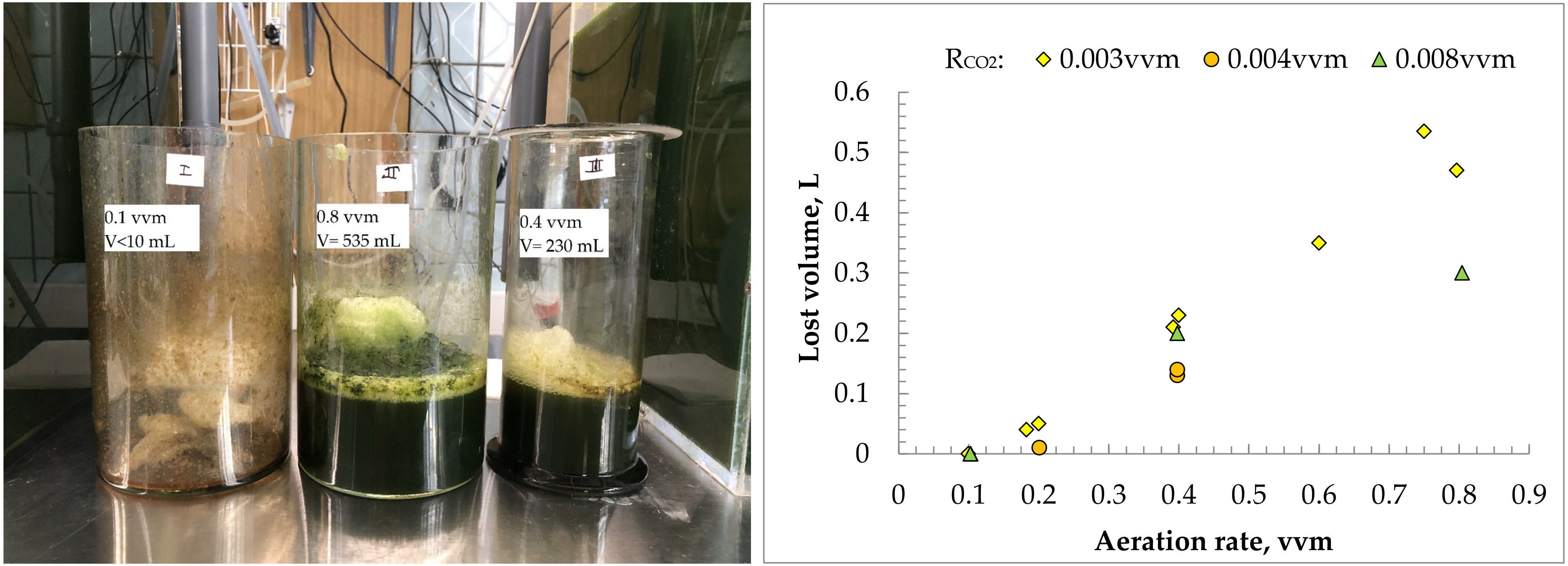

(a) **(b)**

Figure S4. Effect on cultural volume lost by foaming process: (a) – foam containers with lost biomass under different R_{GAM} (0.1, 0.4, 0.8 vvm) and the same R_{CO2} of 0.003 vvm; (b) – dependence of lost volume on the aeration rate (R_{GAM}).

Table S1. Growth characteristics of *C. sorokiniana* IPPAS C-1 cultures grown for 3 days at different temperatures in laboratory system for intensive cultivation. R_{GAM} = 1 vvm, GAM CO_2 concentration 1.2-1.8% , I_{ave} = 400 μmol m^{-2} s^{-1}

Temperature	μ, h^{-1}	T_{dbl}, h	P_{sp}, g dw L^{-1} d^{-1}
26°C±1°C	0.064	10.8	1.1
31°C°±1°C	0.069	10.1	1.5
36°C±1°	0.069	10.1	1.5
41°C±1°	0.047	14.8	0.32

Table S2. Comparative table of growth characteristics of *Chlorella sorokiniana* IPPAS C-1 in FP-5 and FP-18 during 3 days of cultivation.

PBR	R_{CO2}, vvm	R_{GAM}, vvm	n_{CO2}, %	ϱ_0, g dw L^{-1}	ϱ_{fin}, g dw L^{-1}	μ_{total}, h^{-1}	CUE, %	P_{sp}, g dw $L^{-1}d^{-1}$
FP-5	0.003	0.1	3[1]	0.10	3.73	0.051	29	1.21
	0.003	0.2	1.5	0.16±0.00	4.70±0.21	0.047±0.000	35±2	1.51±0.07
	0.003	0.4	0.75	0.15±0.04	3.83±0.26	0.043±0.000	29±2	1.22±0.10
	0.003	0.77	0.36	0.11±0.01	3.73±0.03	0.050±0.001	30±0	1.20±0.01
	0.007	0.45	1.52	0.15±0.06	5.08±0.00	0.051±0.006	17±1	1.64±0.02
	0.008	0.1	7.9	0.13±0.03	3.15±0.48	0.045±0.005	9±1	1.00±0.17
	0.008	0.2	4[1]	0.19	4.30	0.044	12	1.36
	0.008	0.4	2[1]	0.16	4.77	0.047	13	1.53
	0.008	0.8	1[1]	0.14	4.89	0.049	14	1.57
	0.004	0.2	2	0.18±0.01	4.13±0.17	0.043±0.000	22±1	1.31±0.05
	0.002	0.2	1	0.17±0.02	2.49±0.14	0.038±0.001	28±1	0.77±0.04
FP-18	0.003	0.2	1.55	0.16±0.01	3.22±0.02	0.042±0.001	23±1	1.05±0.01
	0.0045	0.23	2	0.10±0.01	2.63±0.06	0.045±0.001	13±0	0.86±0.02
	0.0045	0.45	1	0.09±0.01	3.60±0.07	0.052±0.001	19±1	1.17±0.01

[1] Combination 3% and R_{GAM} of 0.1 vvm was applied in one biological repetition. Also, all experiments under R_{CO2} = 0.008 vvm were made in one biological repetition except 7.9%.
